# On the Application of Microfluidic-Based Technologies in Forensics: A Review

**DOI:** 10.3390/s23135856

**Published:** 2023-06-24

**Authors:** Hanieh Bazyar

**Affiliations:** Engineering Thermodynamics, Process & Energy Department, Faculty of Mechanical, Maritime and Materials Engineering, Delft University of Technology, Leeghwaterstraat 39, 2628CB Delft, The Netherlands; h.bazyar@tudelft.nl; Tel.: +31-152782760

**Keywords:** microfluidic technology, forensic serology, genetic profiling, drug analysis, explosive analysis

## Abstract

Microfluidic technology is a powerful tool to enable the rapid, accurate, and on-site analysis of forensically relevant evidence on a crime scene. This review paper provides a summary on the application of this technology in various forensic investigation fields spanning from forensic serology and human identification to discriminating and analyzing diverse classes of drugs and explosives. Each aspect is further explained by providing a short summary on general forensic workflow and investigations for body fluid identification as well as through the analysis of drugs and explosives. Microfluidic technology, including fabrication methodologies, materials, and working modules, are touched upon. Finally, the current shortcomings on the implementation of the microfluidic technology in the forensic field are discussed along with the future perspectives.

## 1. Introduction

Forensic investigations cover a wide range of diagnosis spanning from identification and analysis of body fluids to drugs of abuse and explosive residues. For each category, well-developed presumptive and confirmatory tests have already been established, which enable forensic investigators and police forces to develop a chain of events and, more importantly, identify the engaged individuals. To find and apprehend any suspects, time may be of the essence. According to Addington, “murders are solved quickly or not at all” [1]. The chance of solving a case normally drops significantly after one week. This further clarifies the need for prompt on-scene identification combined with a qualitative and quantitative analysis of samples during the first hours of investigation, i.e., “golden hours” [2]. Microfluidic technology can address these needs due to distinctive properties, namely portability (small footprint), requirement of small volumes of precious sample, and flexible design providing instant sample analysis. This technology further provides the possibility of (1) integrating multiple analysis steps and (2) analysis of multiple analytes (multiplexing) in a single platform contributing to effective case scenario developments. There are multiple review articles in which the application of microfluidics in specific areas of forensic diagnosis, namely DNA analysis and on-scene human identification [3,4] as well as drug analysis [5], are detailed. In a review paper by Musile et al., applications of a subclass of microfluidic platforms (paper-based microfluidic devices) are discussed for DNA, drug, and explosive analyses with a specific focus on fabrication strategies [6].

This review paper aims to provide a comprehensive overview on “forensic investigations (Section 2)”, “microfluidic technology (Section 3)”, and “microfluidics in forensic applications (Section 4)”. In Section 2, an abridged overview on the current state-of-the-art presumptive and confirmatory methods for forensic serology (detection and identification of body fluids, namely blood, semen, and saliva) is given. In this section, the most encountered classes of drugs and explosives along with the corresponding presumptive and confirmatory methods are further introduced. In Section 3, a general summary on the microfluidic technology, applications, fabrication methods, materials, and various modules which exist in a microfluidic platform are provided. To bridge these two parts, in Section 4 the application of microfluidic technology in various categories of forensic investigations are outlined. These categories include forensic serology, genetic profiling and human identification, screening and analysis of drugs (seized drugs and drugs in biological samples), and analysis of explosives. Finally, the shortcomings of the microfluidic technology for forensic applications, future considerations, and perspectives are provided in Section 5.

## 2. Forensic Investigations

### 2.1. Forensic Serology

Body fluids are among the most vital pieces of evidence found at a crime scene. In serological analysis, evidence is sampled and tested for the presence of body fluids (BFs). The detection and identification of body fluids recovered from a crime scene provide contextual information for forensic casework such as event timeline, scene reconstruction, and involvement of possible individuals [4]. The detection (determining the presence or absence of body fluids) and identification of BFs (determining the source to conclusively identify an individual via further laboratory testing including DNA analysis) are vital in a wide range of investigations [7].

Identifying the nature of a fluid is not always straightforward as many BFs share the same appearance and are invisible to the naked eye [8]. Further absolute confirmation is always necessary for the evidence to be court-proof. The most common BFs discovered at crime scenes are blood, semen, and saliva. Other BFs such as vaginal fluid, sweat, and urine are also essential evidence since they contain valuable genetic information (e.g., DNA). Traditional techniques that are currently used for forensic identification of BFs are categorized into presumptive and confirmatory tests [7]. Presumptive tests are preliminary indicators which can establish the likelihood of the presence and/or absence of a certain BF. Confirmatory tests are utilized to conclusively specify a certain biological material/substance in the BF. Both tests are intended to save time and money via prioritizing samples for further DNA analysis [9]. In review papers by Virkler et al. and An et al., the current and emerging methods for the identification of all BFs are extensively discussed [8,10]. The possible results of the tests can be categorized into (a) true positive (or negative) where the species of interest are (or are not) present and the test outcome indicates a positive (or negative) result; (b) false positive (or negative) wherein the species of interest are not (or are) present, but the test outcome indicates a positive (or negative) result [11]. The advantages of presumptive tests include simplicity, ease of interpretation, narrowing the options for the subsequent test, possibility of utilization on larger areas, and locating evidence not visible to the naked eye. The risk of false positive/negative results, being body-fluid-specific, destructive to genetic evidence (DNA), and not being label-free are among the main disadvantages of most of the presumptive tests [7]. The results of presumptive tests must be supported by confirmatory tests which have a reduced risk of false positive/negative results and can conclusively identify a substance. However, they are costly, time-consuming, non-universal, require additional equipment, and intense sample preparation. Emerging vibrational spectroscopic techniques such as Raman spectroscopy have the potential to provide a universal, non-destructive, and label-free technique for body fluid identification (BFID) at crime scenes [12,13,14]. Raman spectroscopy is intrinsically a very selective and non-destructive method which can preserve the DNA evidence of the tested BF. It has thus gained increasing attention among forensic scientists over the last decade. For instance, coupling Raman spectroscopy with advanced chemometric analysis showed the possibility of determining a donor’s sex as well as race from saliva and blood stains, respectively [15,16]. Using this coupled technique, a differentiation between human and animal blood is also possible [17,18]. This method, based on pairing Raman spectroscopy with chemometrics, can identify and discriminate six forensically relevant BFs, namely blood, semen, saliva, sweat, vaginal fluid, and urine [7,15]. Via Raman spectroscopic mapping on different BFs, a spectral data sheet library is generated. Subsequently a classification model known as “support vector machine discriminant analysis” is developed during the analysis which can achieve a 100% accuracy in the validation step. For more information on this method, the reader is referred to the published works of the Lednev research group at the University of Albany, USA [7,15].

#### 2.1.1. Blood

##### Presumptive Tests

A good presumptive test for blood should be sensitive, quick, simple, safe, and specific [19]. Blood presumptive tests should detect a certain blood component which cannot be commonly found in everyday environment/chemicals [20]. Most presumptive tests for blood achieve this goal based on the peroxidase-like activity of hemoglobin [20]. Blood presumptive tests can be classified into two general categories, i.e., catalytic color tests and forensic (or alternative) light source. Catalytic tests are the largest group of blood-presumptive tests.

All catalytic tests share a similar mechanism at the early step, namely the oxidation of the reagent by a peroxide (e.g., hydrogen peroxide) in the presence of a catalyst (e.g., peroxidase). As stated before, hemoglobin triggers the oxidation of the reagent due to its peroxidase-like activity. These tests are based on peroxidase-catalyzed oxidation in which the oxidation state of a reagent is changed, leading to a color change in the substrate (reagent) [21]. A subcategory of catalytic color tests is chemiluminescence, in which, upon reaction of the reagent with hemoglobin, a light is emitted. In this case, the reagent is electronically excited leading to a subsequent emission of light instead of a color change [21,22]. The production of light is through luminescence and does not require an alternate light source (ALS) [23]. Fluorescein is another common presumptive test which, upon reaction with hemoglobin, produces light. A light source with a wavelength of 415–480 nm (blue light in the visible spectrum) is required to excite fluorescein [24]. In forensic light source or alternate light source (ALS), no chemical reaction is happening and thus it is the safest technique for the investigators. An example is Polilight^®^, in which the suspected area is simply illuminated via a bright light with an adjustable wavelength. The reflected light or the emitted fluorescence (when ultraviolet (UV) wavelengths are used) are an indication of the blood stains [21]. Table 1 summarizes the widely used presumptive tests for blood.

##### Confirmatory Tests

After a positive presumptive test, confirmatory tests should be performed to identify the substance with the lowest likelihood of a false positive [24]. The confirmatory tests can be categorized as follows:

I- Crystal tests (e.g., Takayama and Teichman tests)

The working principle is based on the formation of crystals from heme (an iron-containing prosthetic group which helps the hemoglobin protein function properly). Since other proteins also use heme (such as catalases and peroxidases), they could lead to false positives.

II- Immunochromatographic tests (e.g., HemaTrace^®^, RSID, ELISA)

The working mechanism is based on liquid chromatography in which molecules are separated according to their speed of transport through a liquid. In these tests, a solid phase is also present which uses antibodies. Antibodies are proteins that recognize the shape and characteristic of a biological substance. The immunoassay technique is based on a specific antibody which selectively binds the molecule of interest (antigen). Subsequently, an antibody–antigen complex is formed, which includes a label and can be detected using florescence, for instance. The binding between antibody and antigen is a resemblance of immune system response in which antibodies are generated and bind to antigens (invaders) to remove them [25]. Upon mixing an antibody and antigen in the right ratio, a lattice called precipitin is formed. Cross-reactivity (antibody binds to two or more antigens) can be problematic, leading to false positives. A selective antibody is the one which reacts with very few antigens. In rapid stain identification for blood (RSID-blood), an antibody which recognizes glycophorin (a protein found in erythrocytes (red blood cells)) is used. HemaTrace^®^ uses an antibody which recognizes hemoglobin. Enzyme-linked immunosorbent assay (ELISA) identifies blood based on a similar approach using different antibodies [8]. Immunochromatographic tests generally require specific buffer solutions for the elution of the biological sample. If the BF consists of various materials (e.g., saliva or semen), the application of different tests can be troublesome. Recently, Basset et al. developed a protocol for performing three immunochromatographic tests using the same buffer [9]. RSID is a lateral flow test strip composed of (1) a membrane component enclosed in a plastic cassette, (2) a sample well, and (3) a visualization window. It provides qualitative results as positive or negative depending on the presence or absence of a red or blue line upon adding the sample to the sample well. RSID reader systems have been developed to record and report the results [26].

III- Microscopic tests (e.g., scanning electron microscopy (SEM))

This method concerns the direct visualization of blood cells under a microscope. The visual identification of blood components, such as red and white cells as well as fibrin, is a conclusive proof establishing the presence of blood [8]. SEM imaging of non-conductive materials generally requires the coating of the material with a conductive layer. Coated bloodstains can be imaged at high vacuum mode and high accelerating voltage, leading to high resolution and thus a high level of surface details. When a high level of surface details is not needed, high vacuum and low accelerating voltage can be used for imaging non-coated samples [27,28,29]. Environmental (or variable-pressure) SEM, which works at low vacuum levels with high accelerating voltage, is usually used for examining non-coated bloodstains [30,31]. Advanced light microscopy techniques, such as confocal laser scanning microscope (CLSM), have been recently utilized for imaging bloodstains [32]. CLSM images can provide similar surface details compared to the SEM images of non-coated bloodstains taken at high vacuum and low accelerating voltage. Examining bloodstains on relatively large objects, e.g., household items, using conventional SEM is not practical due to the size constraints of the sample chamber. Light microscopy, e.g., CLSM, does not have the sample size constraint to the same extent as the SEM imaging does. In the case of CLSM, the sample size is dependent on the XYZ range of the motorized stage, which is larger than the conventional SEM chamber [33].

IV- Spectroscopic tests (e.g., ultraviolet-visible (UV–Vis) spectroscopy)

UV–Vis absorption is a reliable technique for confirming the presence of blood. The working mechanism is based on the characteristic absorbance band of various derivatives of hemoglobin, which makes it possible to distinguish between these different derivatives. This absorbance band which is around 400 nm is called Soret band [34]. This method is useful for the identification of older stains which show negative results via presumptive or crystal tests [8]. Environmental conditions, e.g., exposure of the bloodstain to sunlight, heat, or rust interferes with the UV–Vis spectral results. Fluorescence spectroscopy is another method which is based on fluorescence of hematoporphyrin upon excitation with UV light. This method is not affected by the exposure of the bloodstain to environmental conditions and is useful for the detection of old bloodstains on oxidized surfaces [8].

V- Chromatographic tests (e.g., LC–MS/GC–MS)

Examples of these methods include liquid chromatography–mass spectrometry (LC–MS) and/or gas chromatography–mass spectrometry (GC–MS), which are based on the separation of hemoglobin and its derivatives to identify blood. These methods are normally time-consuming since they involve multiple steps and require sample preparation. For more detailed information, the reader is referred to the review paper by Virkler et al. [8].

VI- Methods based on mRNA

These confirmatory tests for blood utilize messenger RNA (mRNA) as, in many proteins, specific sequences of mRNA can be found in high quantity [8,24]. The first step in these methods is the fabrication of a DNA copy of the RNA in the bloodstain using reverse transcriptase [24]. Subsequently analogous to the common DNA profiling, PCR (polymerase chain reaction) is used to amplify the DNA copy. Due to the functional differences between cells and tissues, mRNA markers can be used to identify the most forensically relevant body fluids [35]. In the case of blood, a reliable differentiation between menstrual and non-menstrual blood can be obtained [36]. This method should be used with caution as it can lead to false negative results. Defining cut-off values for the control markers and quantifying corresponding PCR results can be implemented to address the shortcomings [35]. One of the major challenges of this method is RNA degradation and fragmentation after death, leading to a reduction in overall RNA. The results by Bauer et al. revealed that suitable mRNA for reverse transcription PCR (RT-PCR) can still be obtained after post-mortem intervals of around 96 h [37]. mRNA can also be extracted from dried stains as old as 15 years [38]. The condition of storage is important as unfavorable conditions affect RNA more than DNA [35]. The size of the sample is also a crucial parameter; while small-sized samples can lead to results in DNA testing, they may not contain enough RNA for analysis. For more detailed information on the principles, techniques, and applications of RNA analysis in forensic science for BFID, the reader is referred to the review paper by Bauer [35].

#### 2.1.2. Semen

##### Presumptive Tests

I- Alternate light source (ALS) (UV light and Polilight^®^)

It is a non-destructive method for the identification of semen among other BFs. Some light sources such as Wood’s lamp is not very specific and can lead to false positives if ointments or creams are also present [8]. Blue-maxx^TM^ is another commercial light source which has more sensitivity to semen stains [39].

II- Seminal acid phosphatase test (SAP or Aka Walker test)

It is the most popular and accepted presumptive test for semen. Acid phosphatase is an enzyme secreted by the prostate gland, which exists in large amounts in semen [10]. This enzyme can catalyze the hydrolysis of organic phosphates [40]. The product of this reaction reacts with diazonium salt chromogen causing a color change [41]. There can be false positives with the presence of vaginal acid phosphatase. To avoid this, the color change occurring between 5 and 30 s should be considered, as SAP does not give fast results [8]. Currently, Phosphatesmo KM rapid test strips are used for the presumptive testing of acid phosphates. These stripes contain the necessary components which do not require the addition of chemical reagents [42].

III- Microscopy (SEM)

The microscopy technique is not as popular as SAP tests for semen identification. Equipped SEM with an energy dispersive X-ray analyzer (EDX) can be used to detect solidum, chlorine, phosphorous, or metal elements in semen. The proportion of these elements vary among various BFs, leading to the identification of unknown stains. This method is considered as a presumptive test for semen due to the interference of the substrate spectrum which can dominate that of the fluid [8].

##### Confirmatory Tests

I- Microscopic identification of sperm cells using Christmas tree stain

It is a widely accepted confirmatory test for semen since semen is the only BF containing sperm cells. In this method, the sperm cells are made visible via treating the heads (which contain large amounts of DNA) with a stain. The Christmas tree stain is the most popular one, which makes the heads red and the tails green [8].

II- Prostate-specific antigen (PSA) detection

PSA is a glycoprotein produced by prostatic epithelial cells [9]. This antigen is present in seminal plasma and its concentration in other BFs (e.g., urine and breast milk) is very low, eliminating false positives [43]. Immunoelectrophoresis, which involves the combination of diffusion and electrophoresis (movement of charged particles in an electric field), or ELISA are among the original techniques for detecting PSA [8,24]. A commercial kit which works based on antibody–antigen reactions is the Biosign^®^ PSA test, which is cheaper and easier to operate than ELISA [44].

III- Immunochromatographic stripes (e.g., RSID^TM^-semen)

Similar to RSID-blood, RSID^TM^-semen is a lateral flow immunochromatographic test which utilizes two human semenogelin monoclonal antibodies to detect the presence of semenogelin (a protein produced by seminal vesicles) [45]. Compared to other methods, RSID^TM^-semen is faster and has higher sensitivity and specificity as it is specific to human semen only.

IV- mRNA markers

The same method which has already been mentioned for bloodstains based on mRNA can be used for the detection of semen as well [8,35]. Semen-specific markers, e.g., Protamine 1 (PRM1), have been investigated by Alvarez et al., which can be used to detect semen based on RNA and DNA co-isolation [46].

#### 2.1.3. Saliva

##### Presumptive Tests

I- Alternate light source (ALS)

Similar to blood and semen, ALS can also be used to locate saliva stains. The lack of solid particles in saliva makes this technique less straightforward for the identification of saliva compared to the other two BFs [41]. Under UV light, saliva stains show a blue/white color. It is, however, not easy to be distinguished from other BFs. Laser light sources are other examples which have been investigated by Auvdel in 1988 [47]. The Lumatec^®^ superlite 400 (with an emitted light wavelength between 320 and 700 nm) was used to detect human saliva samples on different substrates with different colors. The saliva stains could be detected in darkness or in the presence of daylight [48].

II- Starch-iodide test

This test, as well as the phadebas test, are based on the activity of amylase (the most characteristic enzyme) in saliva regardless of the source of the saliva. In general, the reaction of iodine with starch develops a dark blue color. However, the components of starch (monosaccharides or disaccharides) do not react with iodine and no color will be developed upon the reaction. Amylase in saliva can break down starch into monosaccharides or disaccharides, leading to a color change [8]. This test can give false positives with the presence of other proteins such as albumin and globulin in blood and semen since they can also break down iodine.

III- Phadebas test

The Phadebas reagent consists of a cross-linked starch with a dye (procion red amylopectin). Since amylase can digest starch, the presence of saliva leads to the digestion of starch and thus the release of the dye. The solution turns blue and the intensity of the color can be used for the qualitative and quantitative confirmation of saliva [49]. This test can lead to false positive results in the presence of washing powder, urine, and hand creams [50].

##### Confirmatory Tests

Many methods that have been discussed for blood and semen can also be applied for saliva. Immunological methods such as ELISA have been tested to detect the activity of amylase in saliva stains [51]. Like semen, microscopy techniques, such as SEM-EDX, have also been studied for measuring the relative concentration of potassium, sulfur, phosphorous, sodium, and metal trace elements in saliva samples. Potassium gives the largest peak, which forms the basis of the saliva identification [52]. Fluorescence spectroscopy has also been utilized to detect dried saliva after dissolving the content in potassium chloride (KCl) solution [53]. Emission spectral measured in the range of 345-355 nm showed good intensity.

I- RSID-saliva

A lateral flow immunochromatographic test kit is based on two antisalivary amylase monoclonal antibodies, which can specifically identify human salivary amylase rather than detecting enzyme activity [54]. Like RSID-blood and semen, the method is based on the principles of antibody–antigen interactions. With the presence of saliva, a pink line appears in the observation window [49]. This method has high sensitivity and specificity to human saliva with no cross-reactivity with other BFs, e.g., semen, urine, or vaginal fluid [54]. The extraction buffer can effectively extract amylase from the stain and can detect saliva as low as 1 μL.

II- mRNA markers

Saliva contains specific protein coding genes (statherin (STATH) and histatin 3 (HTN3)) that can be exclusively identified. mRNA profiling is a useful method for saliva stain detection [10]. In the review by Bauer, the use of RNA for the detection of saliva has been discussed [35]. The same co-isolation method for DNA and RNA described by Alvarez et al. is also applicable to saliva stains to detect HTN3 [46]. Saliva-specific genes were also detected by RT-PCR [55]. Similar sensitivity and less specificity, compared to the same method for blood and semen, were obtained.

### 2.2. Forensic Analysis of Drugs and Explosives

#### 2.2.1. Drug Analysis

Forensic drug analysis includes the detection and identification of a suspected controlled substance, e.g., illicit drugs or drugs of abuse. Table 2 shows the classes of commonly used drugs according to certain common effects on the user [56].

##### Presumptive Tests

Presumptive tests for forensic drug analysis identify the presence or absence of certain substance(s) or classes of drugs. These tests include color/spot tests, microcrystalline tests, ultraviolet spectroscopy, thin layer chromatography (TLC), immunoassays, and urine dipstick test [25]. In the following subsections, some of these techniques are explained.

I- Color/spot tests

This presumptive colorimetric test is based on a chemical reaction between a substance (analyte) and an indicator (reagent), which creates a color stain depending on the tested substance. The color spots are visually inspected (by the human eye or color-identification smartphone applications [57]) and compared to a standard color chart (Munsell color chart) [25]. There are many indicators, e.g., nitric acid, marquis, Duquenois–Levine, cobalt thiocyanate, ferric chloride, Dille–Koppanyi, para-dimethylaminobenzaldehyde, potassium permanganate, and silver nitrate [58]; while this method destroys the sample, it is rather sensitive (with a sensitivity limit in the μg range) and specific provided that the proper standards are used. Most drugs of abuse, including analgesics (e.g., opioids), stimulants (e.g., amphetamines, cocaine), plant-based narcotics (e.g., heroin), and psychotomimetic (e.g., lysergic acid diethylamide (LSD)) can be detected with colorimetric tests. For most novel psychoactive substances, associated tests are not there yet [25].

II- Microcrystalline tests

In these tests, upon reaction of a specific reagent with an analyte, unique microcrystals are formed. The evaluation of the formed crystal under normal optical microscope and the comparison with the reference standards are used to identify and detect the substance [59]. Common drugs of abuse, e.g., heroin, methadone, cocaine, methamphetamines, and amphetamines can be identified with this method. It is a specific technique due to the unique choice of reagent and analytes. However, contaminant or dilutents can impede the formation of distinctive microcrystals, limiting this method to pure or purified samples; while this method is relatively cheap, user-friendly, sensitive (μg range), and requires only small amounts of reagents, it destroys the sample and is not quantitative [25].

III- UV–Vis spectroscopy

In this method, UV light is shined through the sample, leading to a rise in the energy level of electrons. A characteristic UV absorption spectrum is then obtained according to the electronic structure of the molecules. UV–Vis can be used for quantitative and qualitative analysis, which can yield structural information as well [25]. It can be used to detect depressants (e.g., diazepam and barbiturates (phenobarbital)), ketamine, and cocaine hydrochlorides. Li et al. used this technique to accurately discriminate various compounds in a mixture [60]. It is a relatively easy method to use and, in combination with chromatographic techniques, higher specificity and selectivity can be obtained.

IV- Thin layer chromatography (TLC)

In this method, there are two phases, namely the planar stationary and liquid mobile phases. The sample is administrated onto the stationary phase and the mobile phase is passed through due to capillary action. The analyte of interest is absorbed in either of these phases and the corresponding retention time is measured [61]. Various components of a sample travel at different paces depending on the size and affinity to a phase. The components are thus separated, leaving the so-called “plate of spots” [25]. TLC can be used to detect various classes of frequently encountered drugs, including depressants (e.g., barbiturates, benzodiazepines, oxycodone), stimulants (cocaine, methylenedioxymethamphetamine (MDMA) or ecstasy, LSD, marijuana), narcotic analgesics (e.g., opium, heroin, morphine), and psychotomimetic (e.g., marijuana, synthetic cannabinoids). The separation and detection of novel psychoactive substances are difficult to achieve with this method [62]; while the TLC method has sensitivity in the microgram range, and is relatively low-cost and easy to operate, it is not specific to a single compound and should be used in conjunction with other methods (e.g., Raman spectroscopy or colorimetric testing) to increase the specificity [61].

##### Confirmatory Tests

Most confirmatory tests for forensic drug analysis are spectrometry-based methods, namely, mass spectrometry (MS), ion mobility spectrometry (IMS), and infrared (IR) spectroscopy. Techniques such as Raman spectroscopy (an optical method) and X-ray diffractometry (XRD) are other widely used methods. In the following subsections, some techniques and the corresponding pros and cons are summarized. The reader is referred to the review paper by Harper et al. for more detailed information on these methods, corresponding working mechanisms, detectable substances, and operational considerations [25].

I- Mass spectrometry combined with chromatographic techniques

This technique is currently the gold standard in forensic drug analysis [63]. In MS, three steps of separation, ionization, and final detection are performed to determine the exact mass to charge ratio (m/z) of ions. In the review by Harper et al., these techniques are explained in detail [25]. In summary, separation techniques include gas chromatography (GC), liquid chromatography (LC), or capillary electrophoresis (CE). Ionization methods can be categorized into soft or hard methods. The commonly used ionization methods in forensic drug analysis are electron ionization, fast atom bombardment, and direct analysis in real time. Using MS combined with chromatographic techniques, any substance with a concentration as low as attomolar range (10${}^{-18}$) can be detected and identified [64]. It requires small amounts of sample and provides unique properties, e.g., high resolution, specificity, and sensitivity. Major drawbacks of this technique are sample destruction, operational costs, requirement of poisonous/hazardous chemicals, non-portability, and requirement of trained personnel [25].

II- Infrared (IR) spectroscopy

This method is based on the amount of absorbed or emitted IR by a sample versus the wavelength. The corresponding spectrum reveals the molecular functional groups [65]. The IR spectra of pure compounds are distinctive fingerprints that can be used to discriminate compounds from each other. Since all compounds have IR-active vibrational modes, this method can be used for the quantitative and qualitative investigation of almost all compounds using reference spectra. Portable infrared spectrometers exist, which can be used at the point-of-need (PON). However, the interpretation of the results requires expert knowledge of the technician/personnel. The quantification of unknown substances is technically possible but can pose a problem due to a laborious procedure and the requirement of a knowledgeable user with expertise in spectroscopy [25].

III- Raman spectroscopy

In Raman spectroscopy, the radiated laser light is scattered by the sample molecules providing spectral vibrational information based on the plot of shifted light intensity as a function of frequency [66]. By determining the active pharmaceutical ingredients as well as polymorphs (molecules with the same chemical formula but different molecular arrangement),any drug can be identified with this technique [25]. Portable Raman spectrometers have been developed (e.g., TruNarc^TM^ by Thermo Fisher Scientific, Waltham, MA, USA), which can be used at the point-of-need (PON) [67]. Raman spectroscopy is a fast and non-destructive method without the requirement of chemical reagents. It can be used to detect multiple substances (both organic and inorganic) in a mixture without any interference form the surrounding water or moisture medium [25]. This method is capable of quantitative and qualitative analysis, but similar to IR, quantitative analysis is an extensive procedure and requires user expertise. The identification and detection of plant-based narcotics, e.g., heroin, can be difficult and requires proper sample preparation since this substance exhibits strong fluorescence.

IV- X-ray diffractometry (XRD)

In XRD, high-energy X-ray radiation is used to bombard the drug sample. The scattering of the X-ray radiation by the crystalline lattice structure of the sample reveals the spatial structure of the molecules. The angle along with the intensity of the diffracted X-ray are used to obtain the crystalline structure and chemical bonds in the sample. Any solid crystalline or partially crystalline substances can be detected with this technique (powder or pills, e.g., cocaine, ketamine, methamphetamines) [68]. XRD has high sensitivity, due to its sensitivity to the polymorphs and contaminants, and high specificity due to its distinctive diffraction lines (X-ray fingerprint) of substances; while it is limited to solid substances and cannot be used out of laboratory, it is non-destructive and requires small sample amounts without any sample preparation [25]. Since, in this method, highly radioactive X-rays are used, high levels of expertise and training are required for the user.

#### 2.2.2. Explosives

Explosives are a mixture of an oxidizer and a fuel in which the oxidizer provides a source of oxygen to induce a combustion-like reaction in the fuel [69]. Oxidizers may present in either of the following forms: (1) heterogeneous mixture, e.g., ammonium nitrate and fuel oil; or (2) in the same molecule, e.g., trinitrotoluene (TNT) [70]. Fuel sources can be categorized into hydrocarbons (e.g., charcoal, sugar, diesel), elemental fuels (e.g., sulfur, aluminum, magnesium), and energetic hydrocarbons (e.g., nitrocellulose, nitrobenzene) [69]. Stimulants, such as heat, shock, friction, etc., are needed to trigger the explosion without any influence on the energy of the explosion. Table 3 summarizes the three classes of explosives [71].

The detection of explosives concerns two general classes, namely homemade (improvised) and military (commercial) explosives. Improvised explosives can be classified into the following categories:

1- Low explosives such as black powder: They contain inorganic salts in a mixture of oxidizers (e.g., perchlorate or nitrate) and fuel (e.g., sugar, sulfur). A mixture of potassium chlorate (known as flash powder) and metal fuels such as Ba, Sr, or Cu, and nitrate salts creates colored flames/fireworks [72].

2- Fertilizer-based explosives: These explosives consist of AN and UN which can be obtained from fertilizers [73]. Mixture of AN and a fuel (e.g., kerosene or diesel) generates a blasting agent [70].

3- Peroxides: These are dangerous primary explosives which can be initiated by impact, heat, or shock [74,75]. These explosives are based on organic and inorganic peroxides which can be easily synthesized using obtainable products [76]. For instance, a mixture of concentrated hydrogen peroxide and a fuel (e.g., flour or pepper) can be used as an explosive. Cyclic organic compounds such as TATP contain peroxides in their functional groups [77]. Organic peroxide does not show fluorescent characteristics nor UV light absorbance, which makes its detection and analysis troublesome [78,79].

Commercial explosives such as Semtex (consisting of pentaerythritol tetranitrate, plasticizers, cyclotrimethylenetrinitramene (RDX)) and C4 (consisting of RDX, stabilizers and plasticizers) have been used in terrorist attacks [80]. In the following sections, the presumptive and confirmatory detection methods for explosives are summarized.

##### Presumptive Tests

These detection methods are simple, rapid, user-friendly, and inexpensive techniques used for on-site detection and identification of the explosive materials.

I- Explosive detection canines

It is the most common method in which dogs are trained to react to a specific scent (or combination of scents) released by the explosives or narcotics. This method has low specificity as the dogs cannot determine which explosive material is present. In addition, it has high maintenance costs and requires a skilled trainer [81].

II- Analytical instruments

Methods such as ion mobility spectrometry (IMS), Raman spectroscopy, and Fourier transform infrared (FT-IR) spectroscopy are examples of analytical methods which rely on the detection of volatile compounds [82,83,84]; while these methods can be portable, they can be costly and bulky in some cases [85].

III- Colorimetric and immunoassay-based tests

Three categories of colorimetric test kits are available commercially.

1- ETK Five: It relies on liquid reagent to detect explosives. The liquid colorimetric reagents are kept in glass ampules which, upon breakage, supply the reagent to an absorbent paper [86].

2- EXPRAY: In this kit, the chemical reagent is sprayed (e.g., aerosol sprays) onto an absorbent pad which is used for swipe sampling [87]. This kit can only detect the family of nitrate compounds leaving out peroxides, chlorates, or perchlorates.

3- XCAT: This kit consists of a portable colorimetric detector and swipe analysis (e.g., optical inks on detection cards). The cards are inserted into XCAT after swiping the sample area. A Software is used to detect and identify the explosive material [88].

While these techniques provide on-site detection, they are not multiplexed, and multiple tests must be performed to analyze an unknown explosive. Other drawbacks include inability to detect perchlorate, requirement of liquid reagents, and possibility of spilling before use [89].

##### Confirmatory Tests

A common inorganic compound of most explosive mixtures, namely nitrates (NO${}_{3}$${}^{-}$), can be used to detect explosives using analytical methods. The detection of explosives containing oxidizers, e.g., perchlorates (ClO${}_{4}$${}^{-}$), chlorates (ClO${}_{3}$${}^{-}$) or peroxides (O${}_{2}$${}^{-}$), is a more complicated procedure [89]. Since these materials possess a wide range of properties (e.g., various composition, volatility, and polarity properties), a variety of analytical methods can used to identify the associated explosives.

I- LC–MS, GC–MS, and high-performance liquid chromatography (HPLC)–MS

GC–MS and LC–MS are used for the detection of organic compounds (e.g., TNT) [77,90,91]. HPLC–MS has been proven useful for the detection of nitrate ions, chlorite, and perchlorate [92]. Mass spectrometry combined with chromatographic methods can also be used for the detection of inorganic ions, namely AN and UN. Since ammonium and nitrates are commonly encountered ions in the environment, these ions should exist in ion pairs to prove the presence of explosives. To achieve this goal, non-aqueous mobile phases (e.g., crown ethers) should be used to ensure that ions do not dissociate [73]. Crown ethers allow for the detection of both organic and inorganic explosives due to lack of interaction with organic explosives [89]. Combined MS with an electrochemical detector can be used for the simultaneous detection of hydrogen peroxide (H${}_{2}$O${}_{2}$) and organic/inorganic ions. The electrochemical detector is placed prior to the MS [93].

II- Ion chromatography (IC) and capillary electrophoresis (CE)

These two methods have been used to detect inorganic compounds (e.g., AN) [78,79,94]. IC equipped with conductivity detection (to measure electrical conductivity between two electrodes) has been developed to detect ionic species such as chlorate, perchlorate, and inorganic nitrate [89]. Gradients in ion chromatography have been shown to be the best procedure to detect inorganic explosives [95]. In CE, contactless conductivity, or indirect UV detection are used to separate ionic species [78,96].

III- FT-IR and Raman spectroscopy

These methods can be used for the simultaneous detection of organic and inorganic explosives, especially at trace levels [89]. They have been utilized for screening peroxide-based explosives [91,97].

IV- SEM and XRD

Metals are identified and detected using the XRD technique or with SEM coupled with energy dispersive spectroscopy (SEM–EDS) [98,99]. These techniques require extensive instrumentation, which is only available in the laboratory, making them non-portable.

## 3. A Short Summary on Microfluidics

Microfluidic technology is characterized by the precise manipulation of a small volume of fluids (mililiter (10${}^{-3}$ L) to picoliter (10${}^{-12}$ L)) in channels with dimensions ranging from 10 to 100 μm. Two distinctive characteristics of microfluidics are (1) small size and (2) manipulating fluids in laminar flow regime [100]. Owing to the small sizes, higher surface-to-volume ratio, greater surface tension, and improved capillary action can be achieved in microfluidic platforms which can provide an enhancement in the conventional separation, detection, and/or analysis methods [101]. The technology in which laboratory-based methods are integrated in a microfluidic chip is known as lab-on-chip (LOC). Microfluidic technology has attracted increasing attention in various fields due to its unique properties, as summarized below [102].

1- Effectiveness: In point-of-care (POC) devices, the possibility of miniaturization leads to a reduction in the sample size as well as the required reagents. Multiplexing capability makes it possible to perform multiple analyses simultaneously within a single device which contains various microchannels. The channel geometry and overall architecture can be readily adjusted leading to the increased efficiency of the analysis. Compared to conventional diagnostic methods, POC devices can dramatically reduce the processing time form hours to minutes.

2- Easy handling: due to the small footprint (smaller than palm size), microfluidics provide prominent advantages, e.g., portability, accessibility, and ease-of-use. This further leads to much simpler devices which do not require trained/expert users.

3- Cost-effective: Compared to conventional devices, microfluidic-based diagnostic devices offer a reduced cost of the final product due to the diverse range of materials available for fabrication. Not only silicon or glass-based wafers, but also a diverse range of polymeric materials such as poly dimethylsiloxane (PDMS), polymethylmethacrylate (PMMA), polystyrene (PS), polyurethane (PU), cyclic olefin copolymer (COC), and polycarbonate (PC) can be utilized to fabricate microfluidic devices. These polymers are cost-effective and can be processed easily. One of the most cost-effective materials which has been used recently is paper. It is lightweight, biocompatible, and disposable.

A diverse application of microfluidics and various materials that can be used to fabricate microfluidic devices along with the fabrication techniques are summarized in Table 4 and Table 5, respectively.

### 3.1. Portable Microfluidic-Based Devices (PMDs)

In various fields spanning from healthcare and clinical studies to forensic diagnostics, there is a great need for a portable device to achieve in situ (real time) qualitative and quantitative analysis of the sample with minimum intervention. Over the last decade, there has been significant research in developing PMDs via combination with smartphones. The current generation of smartphones are equipped with components such as powerful processors, cameras, and a variety of sensors which, along with features such as high data storage capacity, real-time location tracking (GPS), and wireless connectivity, serve as a powerful digital platform for developing PMDs. To improve the field of view, optical components such as customized lenses can be added to smartphones, making them an optical microscope with various imaging modes [103]. They can be then used as readers to analyze results such as colorimetric, chemiluminescent, or fluorescent data. Smartphone-based devices for monitoring blood pressure/pulse rate, diabetic, and weight management have been already commercialized. In a recent review paper by Beduk et al., emerging applications such as electrochemical and optical sensing (smartphone-based multiplexed sensors) for POC devices have been detailed [104]. A smartphone-based acoustofluidic platform has been developed for enhanced colorimetric detection and evaluation of hemoglobin (Hb) in blood [105]. Red and green fluorescence nanoparticles are used as probes for visual testing and measurement of blood Hb levels.

**Table 5:** Overview of the materials and techniques used for fabrication of microfluidic devices (inputs from reference [102]).

Material Type	Subcategory	Example	Fabrication Techniques ^1^	Pros and Cons
Inorganic	Silicon	Silicon wafer	LIGA (X-ray lithography, micro-molding, electroplating) Anodic/fusion bonding (post processing to close open channels)	Resistant to organic solvent Excellent physical properties Need for clean room Expensive Non-flexible Use of toxic chemicals Limited opacity
	Glass	Glass capillary	Photo lithography Wet/dry etching Anodic/fusion bonding (post processing to close open channels)	Optical transparency Chemical inertness Electrical insulation Biocompatible Cumbersome assembly of capillary-based micro reactors Brittle Need for clean room
Organic (polymers)	Elastomer	PDMS	Soft micromachining (e.g., laser ablation) [106,107] Computer numerical control (CNC) micromachining [108,109] Optical/X-ray/photo lithography	Low cost Optical transparency Biocompatible
	Thermoplastic	PC, PMMA, PU, PS	Soft lithography Hot embossing [110,111,112] Injection molding [113]	Disposable Design flexibility
	Cyclic olefin polymers (COPs)	Cyclic olefin copolymers (COCs)	Micromilling CNC machining Hot embossing [114,115] Injection molding 3D printing	Low water absorptivity Electrical insulating Optical transparency High rigidity Inert to acids/alkalines/solvents
Paper	Pressed cellulosic fibers	Pure cotton-based	Inkjet printing Wax patterning Lithography [116] Plasma/laser treatment Paper origami and stacking (for 3D paper-based microfluidics)	Flexible Biocompatible Cost-effective Disposable Special requirements and chemical treatment to avoid fast degradation

^1^ Fabrication techniques, e.g., CNC, lithography, and hot embossing mentioned for polymer-based microfluidics, are mainly used for fabrication of the required master mold to create negative replicas in the corresponding polymer, e.g., PDMS. In the case of paper-based microfluidics, photolithography is used for initial patterning of the paper. For more detailed information, the reader is referred to the corresponding references.

### 3.2. General Components of Microfluidic-Based Point-of-Need Devices (μPON)

**Figure 1:**
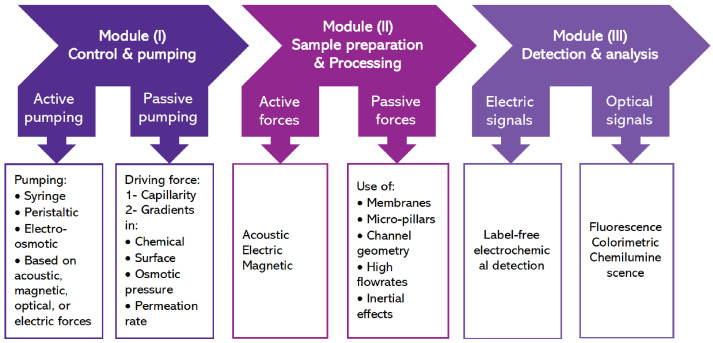
Overview of various modules constituting integrated μPON devices.

An integrated PON microfluidic device consists of three main modules: (I) control and pumping; (II) sample preparation and processing; and (III) detection and analysis. Various microfluidic techniques have been developed to achieve each of these modules [117]. Figure 1 shows an overview of the modules and the corresponding elements which are explained below.

Module (I): Control and pumping

Precise flow control of the sample and reagents is the key to achieve an efficient μPON. Pumping can be categorized into two general classes of active and passive pumping.

1- Active pumping: External forces are applied to drive the flow and control the sample flow rate. In addition to the commonly used syringe and peristaltic pumps, electro-osmotic pumping is another well-known method in which ion drag is established upon application of a tangential electric field leading to a pressure gradient and thus fluid flow [118]. In another method, the digital manipulation of targeted reagents or droplets can be achieved via the utilization of forces such as acoustic [119], magnetic [120], or optical [121].

2- Passive pumping: A driving force inside the microchannels is used to drive the fluid flow without the need for external peripherals. A chemical gradient on the surface, osmotic pressure, capillary gradient, or permeation in PDMS can be used to achieve pumping [122]. The most common passive pumping, which is used in paper-based microfluidics (e.g., lateral flow assays), is the capillary-driven flow [123]. It is a low-cost and simple design without the need for external instruments/power in which the wetting properties of the substrate material are used to drive the flow. The control of the flow rate in capillary-driven flows is achieved through the controlled evaporation of the liquid, or the use of asymmetric micropillars [124]. The major drawback of the capillary-driven flow is the change in wetting properties of the material throughout time, poor reproducibility, and lack of standardization.

Module (II): Sample preparation and processing

In μPON devices, a sample of blood, urine, or saliva should be treated first, i.e., the target analyte must be separated. In some cases, pre-concentration is needed to increase the concentration of the analyte of choice [122]. Similar to pumping, active and passive forces can be used to obtain the desired sorting and separation. An elaborate overview of different approaches for separation/isolation in microfluidic channels can be found in the review by Dalili et al. [125].

1- Active forces: Acoustic, electric, or magnetic forces can be applied in continuous or batch-wise processes for trapping, washing, or enrichment of the cells/analytes. This requires the addition of external or integrated components in the chip. For example, Lenshof et.al. used acoustophoresis to obtain high-quality plasma with low cellular content from the whole blood [126]. They further combined this technique with a silicon-based antibody microarray chip for the detection of a prostate-specific antigen (PSA) via fluorescence readout. Not only silicon, but also PS-based microfluidic channels have been used to implement acoustophoretic-based separation [127]. Techniques based on dielectrophoresis exploit differences in dielectric properties of analytes in a sample to achieve separation [128]. In magnetophoresis, the labeling of analytes with magnetic beads, which have been functionalized with specific antibodies to the target, is required.

2- Passive forces: Inertial effects, altering or modification of geometries within the microfluidic device, incorporating micro pillar arrays or filter membranes can all be used to achieve passive separation [125]. These approaches are more cost-effective as no additional external/integrated peripheral is needed.

Module (III): Detection and analysis of the target

The detection and analysis of the target require the conversion of the biochemical recognition in the analytes into the electrical or optical signals. Dungchai et al. demonstrated label-free electrochemical detection in paper-based microfluidic devices to detect electrically active targets, namely glucose, lactate, and uric acid in biological samples [129]. Optical-based detection methods are mainly based on fluorescence, chemiluminescence, or colorimetric techniques in which the analyte is labeled by attaching a fluorophore or chromophore to an antibody or nucleic acid strand [123]. Colorimetric detection has been widely used in lateral flow assays due to its simplicity and ease of use. In colorimetric detection, a reaction between the molecular probe and the analyte leads to a color change which is visible to the naked eye. The detection of glucose in blood is one of the examples of colorimetric detection [130]. Fluorescence-based techniques make use of fluorescent nanoparticles or quantum dots, demonstrating an increase in sensitivity compared to colorimetric methods. Recently, developments in microfabrication along with advancements in nanotechnology have led to the so-called “nanomaterial-assisted microfluidics” [131]. In these platforms, the multiplexed detection of various biomarkers is possible due to the unique coupling between the microfluidic-based analytical methods and nanomaterial-based biochemistry analysis [131]. Various nanomaterials such as quantum dots, carbon nanotubes, and metal nanoparticles have been implemented to enhance the performance of microfluidic analytical devices, e.g., paper-based and slip-driven microfluidics known as SlipChip [132]. Paper-based analytical microfluidic devices based on SlipChip have also been developed, which are composed of two wax-patterned chromatography paper layers [133]. In a review paper by Wang et al., these highly integrated systems and potential applications in clinical diagnostics are detailed [131]. A combination of nanocatalysis (e.g., enzyme-based nanocatalysts) and microfluidics has also gained attention recently since both components can enable efficient bioanalysis [134]. In a review paper by Gao et al., the recent developments in this emerging field, including widely studied nanocatalysts and microfluidic platforms, detection methods, and unique advantages, are explained in detail [134]. Microfluidic devices with integrated LEDs and microscopes have been developed significantly to detect fluorescence signals.

### 3.3. Microfluidic Paper-Based Analytical Device (μPAD)

To make the microfluidic-based technology for PON analysis widely available, cost optimization is the key element. Paper-based microfluidics can bring down the final product cost due to simplicity, low-cost materials (compared to silicon or glass-based devices), and limited need of external peripherals, e.g., pumps/valves [117]. These devices can be used by non-trained personnel in remote areas wherein resources are scarce and/or laboratories are not available, satisfying the ASSURED criteria: any analytical device must be Affordable, Sensitive, Specific, User-friendly, Rapid and robust, Equipment-free, and Deliverable to enable analysis outside of well-equipped laboratories [135]. μPAD, which was first developed in 2007 by Whitesides group [116], are a subclass of the so-called wicking microfluidic devices in which capillary action is responsible for the transport of the sample and reagent without the need for external peripherals (power source, mechanical components) [136]. Other examples of wicking microfluidic devices exploit membranes (polymeric or glass fiber-based) or cotton threads.

The unique advantages of μPAD such as requirement of low sample volumes, fast results, comparable specificity, and sensitivity to those of immunological assays make them an interesting candidate for diagnosis applications ranging from biomedical to forensics (see Section 4). Such microfluidic devices have been used for blood and urine analysis in medical research [137,138]. Papers used for making microfluidic-based diagnostic devices have special requirements in terms of paper material, porosity, pore size, and wetting behavior [139]:

Paper material: While everyday papers are made of cellulosic fibers obtained from wood, bamboo, or cotton, paper-based microfluidics are made from pure cotton to avoid fast degradation in diagnostic applications.

Porosity: It is the void fraction of the 3D porous structure of the paper which determines the flow rate of the analytes through capillary action.

Pore size: It is defined as the largest diameter of the substances that can pass through the paper.

Wetting behavior: Hydrophilic papers are preferred over hydrophobic ones since most reagents and samples are aqueous-based and thus have more affinity toward hydrophilic substrates. However, patterning the paper into hydrophilic and hydrophobic regions enables multiple assays in a single device since samples can be distributed into several locations [116].

Detection of analytes in paper-based microfluidic devices is based on a reaction between the target analyte in the sample and the reagents (e.g., enzymes, dyes, indicators) which have been already immobilized. Sensing mechanisms that can be implemented are as follows.

1- Colorimetric sensing: Martinez et al. have developed a patterned paper-based microfluidic platform for multiplexed bioassays. Glucose and protein were simultaneously detected in artificial urine samples [116]. The distribution of the sample to various zones led to a color change depending on the type of the analyte. A quantitative measurement of the analyte level was performed by comparing the color to a calibration chart. Colorimetric sensing is the widely used method for the detection of hemoglobin in human blood. Yang et al. developed a μPAD using chromatography paper for Hb detection in blood based on scanning of bloodstains and subsequent digital analysis [140]. A mixture of blood and reagent led to a visible brownish color change which was quantified using a portable scanner. For more detailed information on quantitative colorimetric analysis via μPADs for on-site chemical analysis, the reader is referred to reference [135].

2- Electrochemical detection: A three-electrode paper-based microfluidic platform has been developed by Dungchai et al. for electrochemical detection of analytes in biological samples [129]. Oxidase enzyme reactions in distinct reaction zones were used to detect glucose, lactate, and uric acid. In another study, using commercial handheld glucometer, electrochemical μPADs were developed for quantitative electrochemical analysis of vital biomarkers, e.g., glucose, cholesterol, lactate, and alcohol in human blood [141].

3- Chemiluminescence (CL): It is based on the emission of light upon a chemical reaction. Yu et al. used CL in μPAD to simultaneously detect uric acid and glucose in artificial urine samples [142]. A rhodamine derivative is used to induce CL reaction with the generated hydrogen peroxide. Electrochemiluminescence (ECL) has also been utilized in which luminescence is generated due to an electrochemical reaction. Delaney et al. combined an inkjet-printed paper-based microfluidic substrate with screen-printed electrodes to obtain a sensor to detect 2-(dibutylamino)- ethanol without the need of a photodetector [143]. ECL reaction between Ru(bpy)${}_{3}$${}^{2+}$ and the analyte of choice was used to obtain orange luminescence. Using a mobile camera, the intensity of the sensor luminescence was detected and used to obtain a calibration curve.

## 4. Microfluidics in Forensic Applications

As discussed in detail in Section 2, presumptive and confirmatory tests have some common disadvantages. Most presumptive tests suffer from being (1) body-fluid-specific, (2) prone to false positive/negative results, (3) destructive to valuable DNA evidence, (4) not label-free, and (5) susceptible to sample contamination by chemical reagents. Confirmatory tests, on the other hand, are (1) time-consuming, (2) have a costly procedure, (3) require intense sample preparation, (4) can be destructive, and (5) are non-universal [7,144]. Microfluidic devices and LOC technology (see Section 3) can overcome some of these shortcomings due to distinctive characteristics, namely rapid analysis, decreased volume of reagents/samples, small footprint, portability, reduced risk of (cross-)contamination, and safe storage of sample for further analysis. In the following sections, the application of microfluidics in various categories of forensic analysis is detailed.

### 4.1. Forensic Serology: Body Fluid Screening (BFS) and Identification (BFID)

Body fluid screening (BFS): The determination of the presence of BFs at a crime scene (forensic serology) involves a two-step process in which the potential presence of a BF is first determined via presumptive tests followed by more accurate BFID using confirmatory tests in the second step [145]. In some cases, these multiple tests lack specificity, are time-consuming, can waste the sample, and be destructive to the valuable DNA; while methods based on immunoassay and spectroscopy [8,17,146] as well as advanced ones such as proteomic and epigenetic techniques [147] have been developed with enhanced specificity, they are not portable and require complex devices.

**Figure 2:**
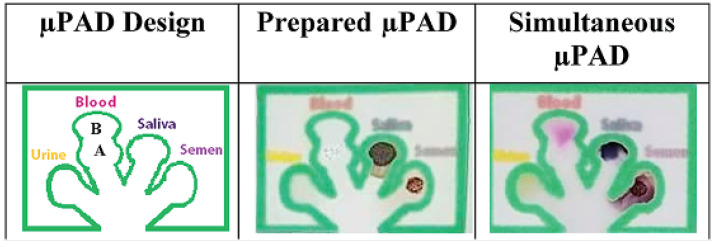
Multiplexed μPAD before and after use in forensic serology for simultaneous detection of urine, blood, saliva, and semen. Sodium perborate tetrahydrate and phenolphthalein are, respectively, placed in areas labeled “A” and “B” [148] (Used with permission of the Royal Society of Chemistry, from (Development of a microfluidic device (μPADs) for forensic serological analysis, Cromartie et al., 11, 5, ©2019); permission conveyed through Copyright Clearance Center, Inc.).

Microfluidic lab-on-chip devices, especially μPADs, can address these shortcomings. Cromartie et al. developed a portable multiplexed μPAD with the colorimetric detection method for presumptive testing of various BFs at a crime scene [148]. This μPAD, which was made of chromatography paper, could simultaneously detect four BFs, namely blood, semen, saliva, and urine. Melted wax was utilized to define hydrophilic channels to guide the sample toward arrays of colorimetric sensors. The biocompatible chromatography paper substrate allowed for the transport of BFs to test wells via capillary wicking. In this μPAD, a single channel branches off into multiple detection pads, creating a branched structure which enabled a multiplex analysis of samples in 10–15 min (Figure 2). The device showed a shelf life of two weeks when stored in a dry and dark location. Traditional colorimetric methods, namely, Kastle–Meyer, starch-iodine, Nesslers reagent, and acid phosphatase were used to detect blood, saliva, urine, and semen, respectively.

**Figure 3:**
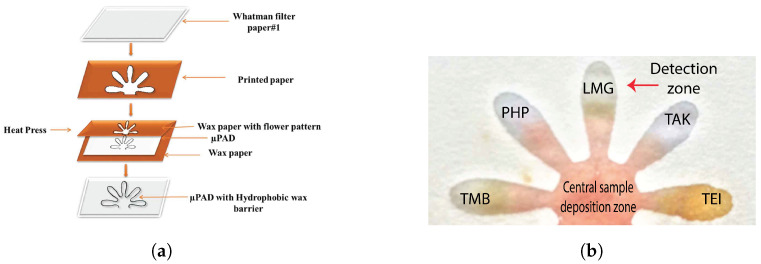
(**a**) μPAD designed using wax printing with a flower pattern containing a single sample deposition and five detection zones. (**b**) Colorimetric detection of blood using different reagents [149] (Reprinted by permission of Taylor & Francis Ltd. (http://www.tandfonline.com (accessed on 12 May 2023)), A portable microfluidic paper-based analytical device for blood detection and typing assay, Ansari et al., Australian Journal of Forensic Sciences, 2021, Taylor & Francis).

μPADs were also used for blood detection and blood typing assays. Ansari et al. developed patterned μPADs using a laser printer and wax for on-site, rapid blood detection and typing [149]. A flower pattern was developed on Whatman paper using wax printing in which the sample deposition zone was connected to five other detection zones (Figure 3a). Routine presumptive and confirmatory tests based on colorimetric reagents, namely TMB, LMG, phenolphthalein (PHP), Takayama (TAK), and Teichman’s (TEI) were used for blood detection on the μPAD (Figure 3b). Dried blood stains (up to 48 days) could be detected with this μPAD.

**Figure 4:**
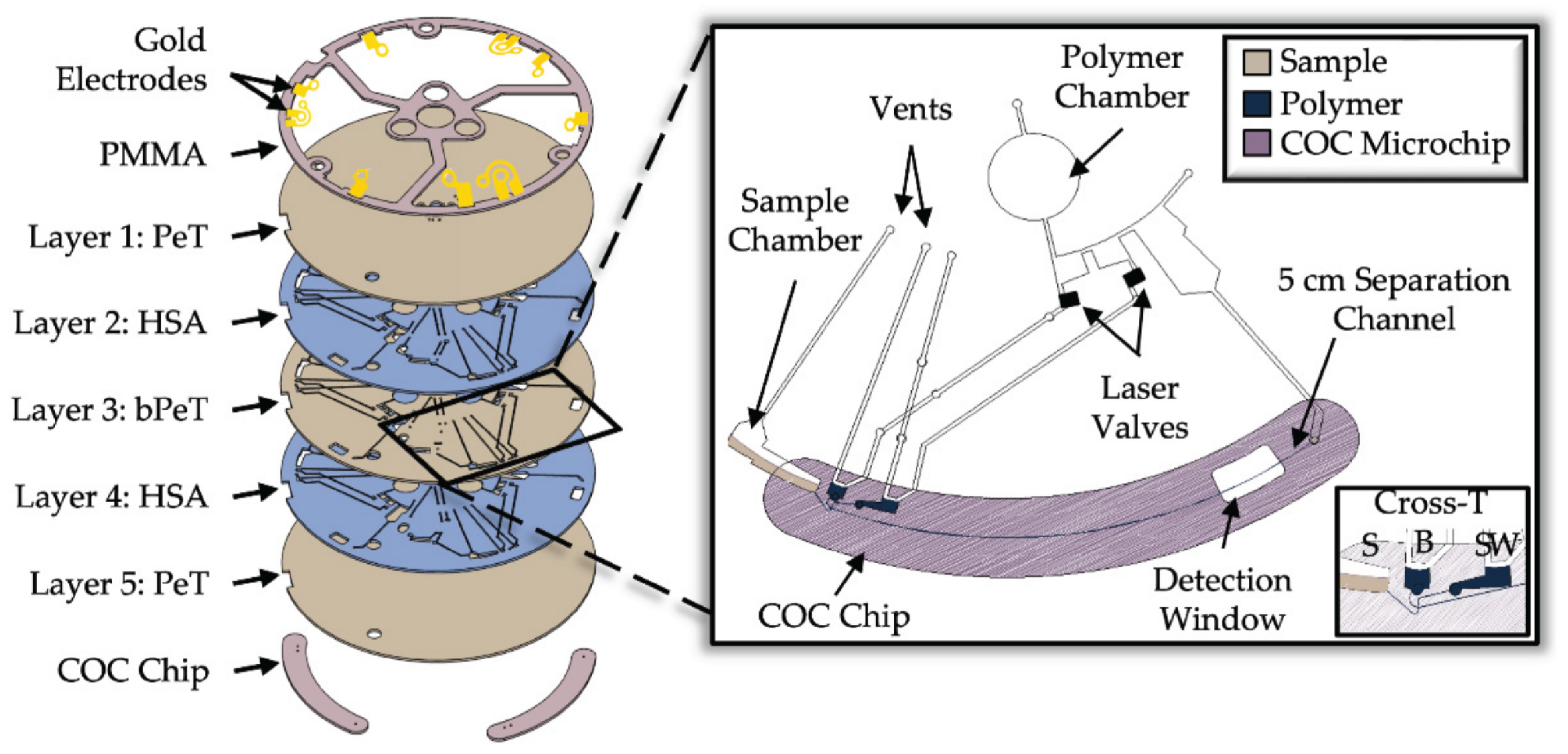
Schematic illustration of the centrifugal microelectrophoresis Disc (μEDisc) for electrophoretic separation of mRNA in various BFs. Microfluidic disc is composed of 5 layers (layers 1–5) made from polyethylene terephthalate (PeT), heat-sensitive adhesive (HSA), black PeT, and PMMA. The separation chip is made from cyclic olefin copolymer (COC) [151] (Copyright ©2022 Layne et al. Published by MDPI. Distributed under the terms of the Creative Commons CC BY 4.0 license, http://creativecommons.org/licenses/by/4.0/ (accessed on 12 May 2023).

Body fluid identification (BFID): current methods for BFID, e.g., spectroscopic-based techniques (Raman or FT-IR), require trained personnel, expensive instruments, and are time-consuming [150]. Emerging methods based on molecular biology, such as transcriptomics, are a promising confirmatory method for BFID since RNA profiling and recovery can be performed on body fluid stains without compromising or consuming the valuable DNA (see confirmatory tests for blood (Section 2.1.1), number 6) [151]. Various types of RNA, e.g., mRNA [152], microRNA [153,154], and circular RNA [155,156] have been investigated. RNA-based BFID involves RT-PCR, which can detect low concentrations of mRNA in small-sized samples [157]. Recently, Layne et al. developed a microfluidic platform (centrifugal microelectrophoresis Disc (μEDisc)) to separate mRNA amplicons in BFs using electrophoresis. A low-cost prototyping technique, namely “print-cut-laminate” [158], was used to fabricate the layered microfluidic platform using various polymers (Figure 4) [151]. PCR-amplified fragments were detected using laser-induced fluorescence. mRNA targets were electrokinetically separated into various BFs, e.g., saliva, blood (menstrual and venous), semen, vaginal fluid, and seminal fluid in pure and mixed forms. Comparable results to those from conventional CE were obtained, but at a four-fold decrease in the analysis time of the electrophoresis.

### 4.2. Genetic Profiling and Human Identification (DNA Typing)

In recent decades, microfluidic technology has been proposed to expedite the current laborious human identification (HID) procedures including DNA typing and short tandem repeat (STR) analysis [159]. Forensic DNA analysis in microfluidics is one of the widely studied applications of microfluidics in forensic diagnosis. This is mainly due to the “Rapid DNA Initiative” proposed by the Federal Bureau of Investigation (FBI) in 2010, which laid the foundation for the integration of microfluidics into existing forensic genetic workflows for HID to obtain an automated and portable device [160]. Over the last decade, multiple reviews have been published in which all aspects of forensic DNA analysis in microfluidics, spanning from methodologies for individual steps, advantages and disadvantages, potential shortcomings, and perspectives have been detailed [3,161]. In a recent review by Bruijns et al., three integrated systems for forensic DNA analysis, which are commercially available (ParaDNA (Laboratory of the Government Chemist, Teddington, UK), RapidHIT(developed by IntegenX which is part of Thermo Fisher Scientific, South San Francisco, CA, USA), and ANDE (developed by NetBio (Waltham, MA, USA))), have been systematically reviewed [162]. Various aspects of these systems, e.g., ease-of-operation, associated costs, time of analysis, and portability are discussed; while the advantages of these systems are prominent, further improvements regarding the possibility of analyzing a wider range of forensic samples and regarding the cost of the cartridges are needed [162]. In another review by Turiello et al., the fully automated microfluidic-based systems for DNA analysis, the so-called, “swab-in-profile-out” have been investigated critically [4]. Despite the tremendous investments and research works which have been performed so far, only a few automated systems are available commercially. The authors further elaborated on the contributing factors as well as technical and contextual reasons for this outcome. There are trade-offs with these automated systems to compete with conventional methods in terms of cost per sample, sensitivity, reproducibility, and multiplexing capability. Here, the process of forensic DNA analysis and application of microfluidics in each step are touched upon. For more detailed information, the reader is referred to individual papers. The five steps of DNA analysis procedure include (1) trace sampling, (2) sample work-up, (3) amplification reaction, (4) detection, and (5) secure storage [3]. The applications of microfluidics in steps (2)–(4) are summarized below.

#### 4.2.1. Microfluidic in DNA Sample Work-Up

DNA sample work-up consists of (1) cell lysis and (2) DNA extraction and purification. The applications of microfluidics in these two steps are summarized below.

1- Cell lysis-on-a-chip: It has been studied based on various lysis mechanisms, e.g., chemical, thermal, electrical/electrochemical, and mechanical [163]. The main advantage of mechanical and electrical lysis over the chemical and thermal one is the absences of reagents and heating elements [164,165]. As an example, Di Carlo et al. introduced the so-called “nano-knives” in the microfluidic channel for mechanical cell lysis [164]. Since only mechanical forces, such as shear and friction, are not sufficient to induce cell lysis, they integrated sharp nanostructures into the channel which could rupture the cell membrane. Other convectional lysis methods including osmotic, optical, acoustic, or ultrasonic have been translated into microfluidic platforms. In a book chapter by Le Gac et al., various cell lysis methods on a chip have been extensively reviewed [166].

2- DNA extraction and purification-on-a-chip: They have been mainly performed by solid phase extraction (SPE) to effectively bind the DNA [167,168,169]. Silica beads are the most widely studied binding agent in microfluidic-based SPE, which can be used in the ng range to achieve efficient DNA adsorption–desorption [170]. Durate et al. introduced the “dynamic SPE” method, in which magnetic silica beads are used on a chip for DNA extraction [171]. Using a small amount of blood sample (0.6 μL), they could recover more than 65% of DNA with concentrations above 3 ng/μL.

**Figure 5:**
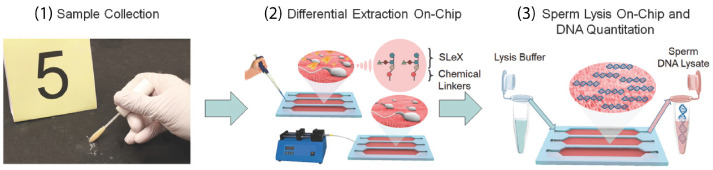
The workflow for on-chip differential extraction used in forensic assault cases to separate sperm and epithelial cells. (2) Single-step pipetting and incubation have been used for sample introduction into the microfluidic chip. Sperm cells are selectively captured in the channels, while epithelial cells are removed due to their lack of adhesion to the channel wall and their large size. (3) Lysis-on-chip using a buffer is utilized for sperm cell lysis and DNA is collected for further genomic analysis [176] (Copyright ©2018 Inci et al. Published by WILEY-VCH Verlag GmbH & Co. KGaA, Weinheim. Distributed under the terms of the Creative Commons CC BY license, https://creativecommons.org/licenses/(accessed on 12 May 2023).

Most of these methods have already been implemented in clinical applications but the application in the forensic field imposes complications due to a wide variety of raw samples which are, in most cases, contaminated by chemical, biological, or radiological agents [172]. A more complicated extraction procedure involves sexual assault cases, in which the sample contains a mixture of cells (epithelial and sperm) from at least two donors [173]. Differential extraction (DE) methodologies have been developed to enable the separation of DNA fractions from both male and female samples. Ultrasound and sonication have been used for successful DE for forensic analysis of sexual assault cases [174,175]. In a short period of 15 min, Voorhees Norris et al. used ultrasound to selectively capture sperm cells from a female epithelia cell lysate [174]. In a patent by Belgrader et al., sonication and filtration were used to lyse epithelial cells and separate them from sperm cells, respectively, [175]. In review papers by Clark et al. and Chong et al., the corresponding dominant methodologies and emerging approaches are discussed in detail [172,173]. Microfluidic technology has shown promising results in terms of analysis time, reliability, accuracy, and ease-of-use for DE applications. Inci et al. implemented a carbohydrate ligand for binding egg and sperm (oligosaccharide sequence (SLeX)) on-chip to selectively capture sperm cells followed by sperm lysis on-chip for further DNA genomic analysis [176] (Figure 5). The method has been validated using forensic mock samples from a decade ago showing 70–92% capture efficiency. It could further reduce the DE analysis time from 8 h to 80 min.

#### 4.2.2. Microfluidics in DNA Amplification and Detection

DNA amplification is an essential step in forensic DNA typing since generally forensic samples have a low amount of DNA. The amplification reaction must be performed to increase the amount of DNA for the subsequent step of detection and STR profiling [3]. Polymerase chain reaction (PCR) is a widely used amplification method which is also one of the most time-consuming steps of DNA analysis. The first application of microfluidics for PCR amplification was introduced by Kopp et al. in 1998 [177]. A wide variety of microfluidic-based PCR methods have been investigated since. Two main types of PCR chips, namely “well-based” and “continuous flow”, have been developed [178]. In the well-based PCR chips, the entire chip goes through thermal cycling, while in the continuous flow PCR chips, the sample is heated/cooled locally via fixed temperature zones.

**Figure 6:**
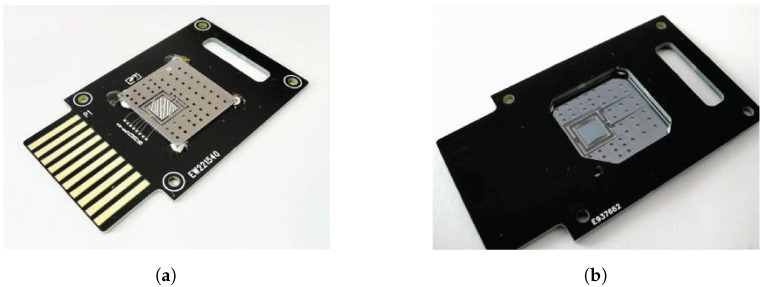
Microfluidic chip for DNA fingerprinting based on combination of PCR amplification and HyBeacon melting assays developed by Cornelis et al. (**a**) Top view of the complete chip with integrated heaters, 24 inlets, and printed circuit boards. (**b**) Back side of the chip showing reaction chambers and access holes connected via microfluidic channels [185] (Copyright ©2019, Cornelis et al. Published by Springer Nature. Distributed under the terms of the Creative Commons Attribution 4.0 Generic License, http://creativecommons.org/licenses/by/4.0/(accessed on 12 May 2023).

Microfluidic-based PCR in droplets is an emerging technique in which each droplet acts as an individual pL-nL sized reactor [179]. Monodisperse water-in-oil droplets can be formed in microfluidic channels via different techniques [180,181]. Droplet-based PCR has unique advantages, namely (1) faster mass transfer and better mixing due to increased surface-to-volume ratio and (2) prevention of (cross-)contamination since droplets are isolated and act as separate reactors [179]. In review papers by Ahrberg et al. and Bruijns et al., a detailed overview on various (real-time) PCR chips including droplet-based, isothermal, and digital ones is provided [3,182]. Multiplexed PCR in which multiple DNA loci are amplified simultaneously has also been studied on microfluidic platforms [183,184]. Estes et al. significantly improved the on-chip PCR via enhancing control systems, valves, software, and amplification chemistry [183]. Via an optimized solid phase encapsulating assay mix integrated within the microfluidic platform, they encapsulated the reagent for PCR into a solid phase with long shelf life, enabling multiplexed PCR on-field. DuVall et al. studied chip-based multiplexed PCR on various substates, namely transparent and black PeT, and obtained STR profiles in 10–15 min (depending on the size of the multiplex) [184]. Their microdevice showed a great potential for integration within upstream DNA extraction as well as downstream electrophoresis. Cornelis et al. developed a novel forensic DNA fingerprinting by combining PCR amplification and HyBeacon melting assays in a single microfluidic chip with integrated heating elements [185]. Four STR loci and amelogenin gender markers could be analyzed simultaneously, showing a step forward for mass-producing portable devices for on-site forensic DNA analysis (Figure 6).

DNA detection techniques on a chip are mainly based on fluorescence sometimes combined with capillary electrophoresis (CE). In conventional CE, a long (circa 30–60 cm) circular silica capillary is used in which amplicons (tagged with fluorescent dye) are injected. Upon application of voltage, the amplicons are separated (based on size) and move across a detection window wherein they are excited by a laser. The detection system and further analysis produce the corresponding electropherogram to accurately size DNA fragments and obtain STR profiles [186]. Due to the simplicity of the capillaries and the associated mechanisms, CE was successfully translated into microfluidics. Microchip electrophoresis (ME) was developed via optically clear materials (glass and transparent polymers), which consisted of circular channels as a reminiscence of silica capillaries [159,187]. Due to reduced lengths in channels in ME, a 10–100 times faster separation could be achieved compared to conventional CE [159]. The integration of detection on-chip is not an easy task and still most of the developed MEs use off-chip detection methods [3]. SYBR Green I or EvaGreen are widely used fluorescent dyes for this application due to their simplicity and fast results [188]. However, they are non-specific and multiplexing is not possible. To achieve specific detection, fluorescent primers or probes should be utilized at the cost of more complicated chip design. Hopwood et al. developed an integrated microfluidic system in which three steps of DNA purification, PCR-based amplification, as well as separation and detection using CE were performed in separate reaction chambers in a single device [189]. A micro CE with a resolution of 1.2 base pairs was used to separate fluorescently labelled STR fragments. The produced DNA profile, which was achieved in <4 h, was compatible with the UK DNA database. A detailed overview on various chip-based detection methods, DNA biosensing, and profiling via STR analysis is given in two reviews by Bruijns et al. [3,190].

### 4.3. Illicit Drugs and Drugs of Abuse

The largest application field of microfluidics in forensic drug analysis is the determination of illicit drugs. It provides accurate quantitative and qualitative data for on-site detection and analysis for cases ranging from drug analysis in sports to driving under the influence of drugs [191]. Various analytical drug detection and analysis techniques, namely screening via immunoassays (e.g., ELISA), extraction (e.g., filtration, SPE), separation (e.g., CE, micellar electrokinetic chromatography (MEKC)), and detection (e.g., CL, electrochemical) (see Section 2.2.1) have been translated into microfluidic platforms and are detailed in a review by Al-Hetlani [5]. The first application of microfluidics for analysis of drugs of abuse dated back to 2000 and was developed by Greenway et al. [192]. In this platform, Ru(bipy)${}_{3}$${}^{2+}$ chemiluminescence (CL) was used for the detection of cocaine.

Drug analysis can be categorized into seized drugs and drugs of abuse in biological samples. The analysis of possible adulteration in seized drugs as well as food and beverages (using psychoactive drugs) is an important forensic investigation topic [6]. In the following subsections, the applications of microfluidics in these two categories are summarized.

#### 4.3.1. Seized Drugs

The presumptive identification of seized drugs is classified into (1) colorimetric and (2) electrochemical analysis [6]. An example of electrochemical analysis includes square wave voltammetry used by Riberio et al. to detect LSD after dissolution in LiClO${}_{4}$ in a paper-based microfluidic device [193]. The colorimetric detection methods are summarized below.

**Figure 7:**
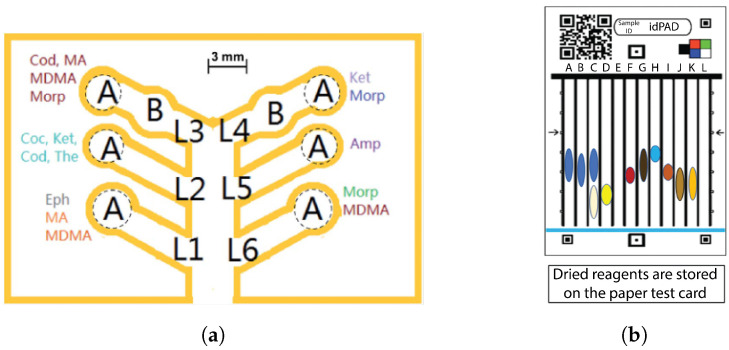
(**a**) Developed μPAD by Musile et al. for multiplexed screening of seized drugs based on colorimetric detection. In each lane, one or two zones are realized for placing of reagents (A, B). Through the middle stem, the sample (dissolved in a solvent) is introduced and travels toward each reaction zone via capillary action. Abbreviation of drug names: Eph (ephedrine), MA and MDMA (methamphetamine), Coc (cocaine), Cod (codeine), Ket (ketamine), The (thebaine), Morp (morphine), Amp (amphetamine) [194] (Used with permission of the Royal Society of Chemistry, from (The development of paper microfluidic devices for presumptive drug detection, Musile et al., 7, 19, ©2015); permission conveyed through Copyright Clearance Center, Inc.). (**b**) The idPAD developed by Lockwood et al. for simultaneous detection of twelve drug compounds [197] (Permission is granted for Lockwood et al., idPAD: Paper analytical device for presumptive identification of illicit drugs, Wiley-Blackwell, ©2020 American Academy of Forensic Sciences).

In 2015, Musile et al. introduced the first μPAD platform for multiplexed colorimetric detection of various drug compounds, namely cocaine, opiates, ketamine, and phenylamines [194]. Six hydrophilic channels were created on chromatographic paper utilizing wax printing and were connected to a single stem. Various colorimetric reagents were placed in each channel, enabling multiplexing (Figure 7a). Krauss et al. developed a centrifugal microfluidic platform (made from inexpensive polyester toner) in which the tested material was placed into multiple reaction chambers for fast screening [195]. An enhanced objective image analysis method (based on hue and saturation analysis) using a smartphone was developed for more accurate colorimetric detection and decreased analysis time (compared to scanner-based methods). As a proof-of-concept, cocaine and methamphetamine were tested with a limit of detection (LOD) in the mg range. The device could identify 30 unknown samples.

To further improve the specificity of presumptive illegal drug testing and overcome the inherent subjective interpretation, Bruijns et al. developed a microfluidic platform using cyclic olefin copolymer (COC) for presumptive testing [196]. A portable UV–Vis spectrometer was combined with the microchip, providing more analytical information about the compound based on the accurate absorption wavelength. Lockwood et al. developed a twelve-lane paper-based analytical device (idPAD) for the detection of a more complex drug mixture (illicit drugs and cutting agents) in seized drugs with 95% sensitivity, 100% specificity, and LOD in the μg range [197] (Figure 7b). The idPAD was printed with wax followed by baking at 100 °C to form a hydrophobic barrier and deposition of reagents into the 12 lanes. After the addition of the sample in the dry form on the “swipe line”, the card was placed for 3 min in water to rehydrate the reagents and bring the chemicals into contact with each other via capillary action. The developed unique color was analyzed and compared with a standard library. The idPAD demonstrates an inexpensive and user-friendly platform for on-field detection of illicit substances (e.g., cocaine HCl, heroin, crack, and methamphetamine) which does not require samples in the solution form.

**Figure 8:**
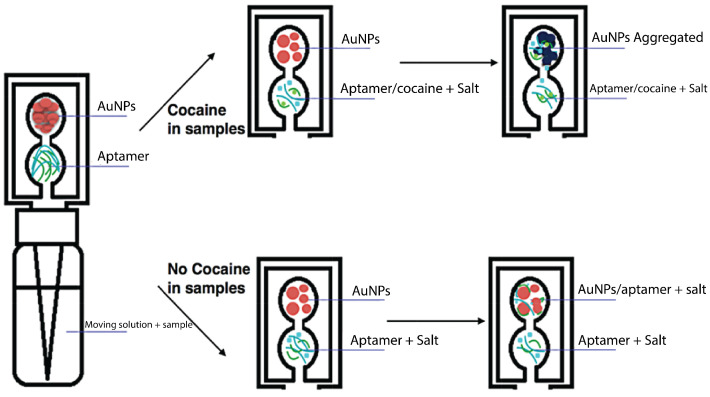
Aptamer-based μPAD coupled with gold nanoparticles (AuNPs) developed by Wang et al. for detection of cocaine in seized drugs. Aptamers and salts are placed in the middle of the channel, while AuNPs are placed at the end. The presence of cocaine and salt leads to the aggregation of AuNPs and thus a color change [203] (Permission is granted for Wang et al., An aptamer-based paper microfluidic device for the colorimetric determination of cocaine, Wiley-VCH, ©2017 WILEY-VCH Verlag GmbH & Co. KGaA, Weinheim).

More selective colorimetric-based detection methods can be achieved using aptamers/ antibody recognition. Aptamers are engineered nucleic acids with specific recognition characteristics for small molecules. Based on ligand binding, the conformation of the aptamer changes, enabling molecular recognition [198]. Cocaine detection using a DNA aptamer has been investigated based on fluorescence [199,200] and electrochemical detection methods [201,202]. Wang et al. developed a μPAD on which gold nanoparticles and anti-cocaine aptamers were coupled for the detection of seized cocaine samples [203]. Gold nanoparticles on the microfluidic paper were aggregated in the presence of salt and cocaine, leading to a color change (black) detectable by the naked eye or a camera (Figure 8). This aptamer-based μPAD provides high specificity, sensitivity, and can detect cocaine in <5 min with LOD in the μg range. With the development of new aptamers, this technique can be used to detect other illicit drugs.

In another study, Kawano et al. developed a highly selective method to detect cocaine in a microchip using a biological nanopore (transmembrane toxin R-hemolysin (αHL)) combined with a DNA aptamer (cocaine-binding aptamer (CBA)) [204]. In the absence of cocaine, the single-stranded CBA with a diameter around 1 nm can pass through the αHL pore which has a larger diameter (1.5 nm). In the presence of cocaine, the ligan-bound aptamer cannot pass through and is captured by the nanopore. The change in the channel currents was used to detect the presence of ligand-bound aptamers and thus cocaine. The device could detect cocaine in <1 min with LOD in the ng range (cutoff limit of the drug test).

#### 4.3.2. Drugs in Biological Samples

Single compound: Microchip-based ELISA has been used for the determination of amphetamine in plasma and urine samples using CL detection [205] and D-methamphetamine in hair samples [206]. Mobioni Far et al. developed a disposable microfluidic chip which consisted of a serpentine channel coated with antibodies [205]. The sample (containing amphetamine) was mixed with labeled amphetamine (with horseradish peroxidase) and pumped through the channel. The amphetamine (unbound and horseradish peroxidase-labeled) was removed from the channel and CL was triggered using luminol and hydrogen peroxide. The concentration of the drug was inversely proportional to the detected signal. A colorimetric hydrogel-μPAD was developed by Tian et al. based on highly selective aptamer-based recognition to detect cocaine in urine samples [207]. Crosslinked hydrogels with glucoamylase-trapped aptamers were used as the molecular recognition unit showing amplified signals upon cascade enzymatic reaction; while the platform showed lower sensitivity with cocaine metabolite (present in urine after cocaine ingestion), it showed high sensitivity to cocaine itself, showing a great potential in detecting cocaine as the major compound in blood/saliva. For more examples, the reader is referred to the review paper by Musile et al. [6].

Multiple compounds: Detecting various drug compounds in a biological sample first requires extraction and preconcentration followed by separation and detection. Liquid–liquid extraction (LLE) was implemented on a microchip by Miyaguchi et al. for the extraction of amphetamine, methamphetamine, methoxyphenamine, and mephentermine in urine samples [206]. Monoliths as a new class of materials have been used for the extraction of pharmaceutical compounds [208]. Monoliths can be formed in situ in microfluidic chips providing a stationary phase for separation. Xu et al. used organic monoliths in a PDMS microchip for the extraction of promethazine in synthetic plasma samples [209]. The microchip consisted of a monolith channel and a detection channel to detect promethazine based on CL.

In forensic drug analysis, separation is normally conducted using electrokinetic-based methods, namely CE and MEKC [5]. Du et al. implemented MEKC separation combined with Ru(bipy)${}_{3}$${}^{2+}$ ECL detection on a chip to separate and detect codeine and heroin in urine samples [210]. Electrophoretic-based separation combined with the UV detection method has been used by Qiag et al. to detect eight illicit drug compounds (from a family of narcotics, depressants, and anti-depressants) in urine samples [211]. A PMMA microfluidic chip was coupled to a printed circuit board to apply the required voltage for separation. The UV detection was performed on-chip using a custom-built set-up. The extraction was performed off-chip prior to separation and detection via LLE. Other separation methods, e.g., HPLC, have also been performed on a chip. Bait et al. developed the first HPLC-on-a-chip using commercial polyimide-based platforms for the detection of flunitrazepam (rohypnol), a drug associated with rape cases, in urine [212]. The metabolite of rohypnol (7-aminoflunitrazepam) was detected in urine samples with LOD in the ng range. Comparison of the on-chip results with the routine GC–MS showed a difference of less than 20%.

### 4.4. Explosive Residues

There is a great demand for the rapid determination of explosives on the field, e.g., near military bases and areas under terrorist attacks. The latter is of utmost importance due to the tremendous increase in the use of improvised explosives by criminals since military explosives are more controlled and not easily accessible [213]. Microfluidics can offer small, portable, and user-friendly platforms for accurate presumptive explosive detection on-site. Over the last decade, colorimetric and electrochemical detection have been among the most widely used methods to detect high and low explosives.

Detection of high explosives: Due to the redox properties of the nitroaromatic explosives, they can be readily detected electrochemically. The inherent compact design of electrochemical detection (ECD) methods along with less signal loss and smaller detection volumes (compared to absorbance- or fluorescence-based methods) make these techniques a suitable candidate for miniaturization and integration on-chip [214]. The first applications of electrochemical detection of explosives on a chip (coupled with CE separation) dates back to 2000 [215,216]. Wang et al. developed a glass-based microchip electrophoresis combined with ECD to detect a mixture of high explosives including TNT [215,217]. They used amperometry [217] and square-wave voltammetry [215] methods as the ECD methods; while the amperometric detection demonstrated higher sensitivity with LOD in the μg range for both TNT and DNB (1,3-dinitrobenzen) [217], the voltametric detection provided more information. In the integrated microfluidic chip (CE combined with ECD (CE–ECD)) developed by Hilmi et al., the working electrode (made from Au) was directly deposited into the separation channel of the CE for amperometric detection [216]. A mixture of four nitroaromatic-based explosives (family of TNT and dinitrotoluene (DNT)) were analyzed in 130 s using a sodium dodecyl sulfate (SDS)/borate buffer. They achieved higher sensitivity with LOD in the ng range owing to the highly active surface area provided by the gold nanoparticles. In the work by Piccin et al., the same integration strategy (CE–ECD)) was used to develop microchip protocols for the fast screening and detection of nitrate ester explosives (including low-temperature PETN (pentaerythritol tetranitrate) as one of the strongest high explosives) [79]. Four nitrate ester explosives could be separated using a 1500 V separation voltage in less than 3 min and amperometrically detected with high sensitivity (LOD in ppm range). The developed microchip showed great promise for onsite threat evaluation and security screening, namely testing people’s luggage, vehicle, or packages for nitrate ester explosives.

**Figure 9:**
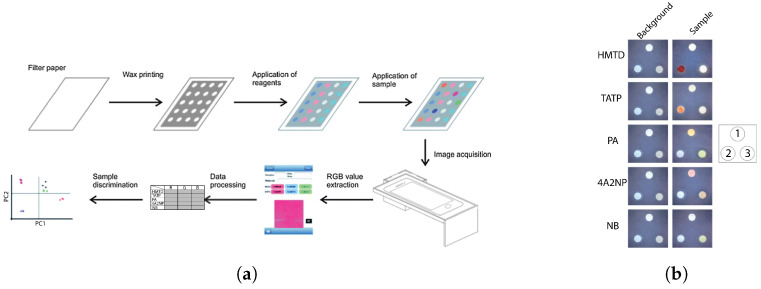
(**a**) The fabrication and measurement process of the μPAD developed by Salles et al. for colorimetric discrimination of (**b**) five high explosives upon reaction with each reagent (1: creatinine, 2: KI/H${}^{+}$, and 3: aniline) (Used with permission of the Royal Society of Chemistry, from (Explosive colorimetric discrimination using a smartphone, paper device and chemometrical approach, Salles et al., 6, 7, ©2014); permission conveyed through Copyright Clearance Center, Inc.) [220].

Colorimetric methods on microfluidic platforms have also been implemented for testing high and military explosives. In 2015, Peters et al., developed a μPAD for the detection of high explosives, e.g., urea nitrate, TNT, and RDX [218]. Explosives including organic peroxide (TATP) and its precursor hydrogen peroxide could also be detected with LOD in the μg range. The μPAD consisted of five lanes, which were fabricated on a chromatography paper using wax printing. The corresponding colorimetric reagent was deposited on each lane showing a color change upon reaction with the explosive after 5 min. In all experiments, a mixture of acetone–water (50:50) was used as the solvent. This platform demonstrated an inexpensive, easy-to-use, and portable test for the rapid identification of explosive residues. Pesenti et al. developed a μPAD consisting of two areas for presumptive colorimetric as well as confirmatory tests for the detection of trinitro aromatic explosives, e.g., TNT and trinitrobenzene (TNB) [219]. The device could detect TNT and TNB with LOD in the ng range with no interference from commercial products, such as perfumes, cleaning agents, oxidizers, etc. Another colorimetric-based μPAD for the identification of high explosives, including TATP, was developed by Salles et al. [220]. Three reagents (KI, creatinine, and aniline) were used to discriminate five explosives based on the unique color pattern upon the reaction between the two, which was evaluated via a smartphone. Using unique analysis methods, namely hierarchical clustering analysis and principal component analysis, the analytes could be readily discriminated after 15 min (Figure 9).

**Figure 10:**
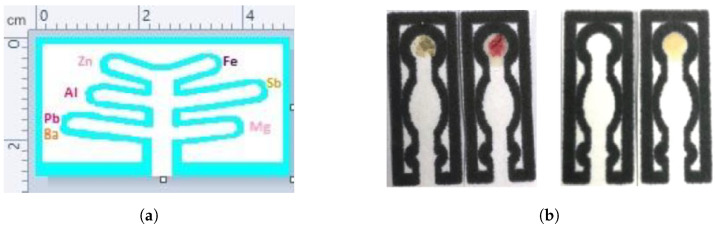
(**a**) The multiplexed μPAD design developed by Chabaud et al. for detection of various metal salts as inorganic residues of low explosives. (**b**) Results of colorimetric test on a single lane showing color change from tan to pink/purple for lead (left) and colorless to brown for antimony (right). In all experiments, water was used at the solvent [221] (Reprinted from publication Simultaneous colorimetric detection of metallic salts contained in low explosives residue using a microfluidic paper-based analytical device (μPAD), 9, 35–41, Chabaud et al., Copyright ©2018, with permission from Elsevier).

Detection of low explosives and blasting agents: μPADs were the most developed microfluidic platforms in the recent years for on-site colorimetric detection of low explosives and blasting agents. Chabaud et al. developed a μPAD for the simultaneous detection of inorganic metallic salts present in primer residues and fireworks [221]. The μPAD consisting of six hydrophobic channels was made by wax printing on chromatography paper. In each channel, a specific reagent was placed, demonstrating a color change upon reaction with the metal salt. The device could detect six metals (lead (Pb), iron (Fe), antimony (Sb), zinc (Zn), aluminum (Al), and barium (Ba)) in less than 10 min with LOD in the μg range (Figure 10).

A five-lane μPAD was developed by Peters et al. for the detection of inorganic explosives, e.g., black powder, flash powder, and ammonium nitrate [218]. The fabrication strategy was similar to the previously described method (wax printing to create hydrophobic channels on chromatography paper). The explosives were dissolved in deionized water to enable capillary action. The μPAD could detect inorganic explosives, e.g., ammonium, chlorate and perchlorate oxidizers, nitrate, and nitrite with LOD in the μg range based on the color change upon reaction with the deposited reagent on each lane.

## 5. The Road Ahead for Microfluidic-Based Forensic Diagnosis

### 5.1. Shortcomings

Microfluidic platforms can serve as rapid and easy-to-use methodologies for on-scene forensic analysis. They are potentially cost-effective, non-destructive, and accurate, depending on the method and corresponding integrated analysis on the chip. Despite these advantages, they are not yet universally implemented in the field of forensic analysis. The possible reasons for this are as follows:

1. Lack of standardization: Some developed microfluidic platforms, specifically paper-based ones, cannot withstand harsh environmental conditions, are sensitive to temperature and/or humidity, show limited stability of chemical reagents, and can have variations from batch to batch [117]. All these factors result in a lack of standardization which further impedes the acceptance of these platforms by the forensic authorities.
2. Challenges in integration: An ideal microfluidic device for on-scene application should provide the so-called “sample-to-answer” and directly connect the forensic investigators to the results. The laboratory-based confirmatory tests (e.g., assays) normally involve multi-step procedures requiring sample collection and processing (e.g., pre-concentration), chemical/biological reactions and generation of signals, detection, analysis, and final reporting of the results. A successful microfluidic device which can provide a rapid and accurate alternative method on-site should have all these steps integrated and automated in a single platform. A vast majority of research on this field has mainly focused on developing proof-of-concept methodologies for individual steps as independent technologies. Undoubtedly, discretization is an imperative stage of developing any technology for resolving potential problems. To realize an end product, however, all these discrete technologies must be integrated. The transition from laboratory microfluidic prototypes to a commercial product is still challenging. Most of these platforms are mainly tested under controlled laboratory conditions, which makes them difficult to integrate with the other technologies under realistic conditions.
3. Product cost: Material and manufacturing methods must be considered for mass production to enable a smooth transition of the technology to the forensic field. Most laboratory-based platforms are made of glass, silicon, or PDMS, which require cleanroom facilities and lithography techniques; while plastic and paper-based platforms are affordable alternatives for mass production, their universal applicability is questionable. The choice of material is highly constrained by the application, compatibility with the sample, and possible integration with detection elements.
4. Associated trade-offs with sample-to-answer platforms: Up to this date, there are few commercial rapid DNA analysis platforms which can provide a sample-to-DNA profile. Compared to the conventional method, these platforms have some limitations and trade-offs including reduced sensitivity, higher costs than originally anticipated, speed, and throughput [4]. These trade-offs along with the cultural forensic landscape have further limited the use of such commercial sample-to-answer platforms, making the implementation of fluidic technology in the forensic field a complex task.

### 5.2. Future Perspectives

Considering the shortcomings and limitations explained above, the following strategies are proposed to further enhance the implementation of the microfluidic technology in the forensic field.

1. Enhancing the existing capabilities: plastic and paper-based microfluidic platforms have grown tremendously over the last decade, mainly due to their low cost and ease-of use. These platforms offer multiplexing for simultaneous analysis of multiple compounds. At this stage, a focus change toward standardization and integration of these platforms with electronic devices (e.g., smartphones for detection and/or analysis steps) can further expand their applicability in different forensic fields.
2. Empowering the current methodologies: as stated above, the integration of all analysis steps in a single platform is challenging, which in some cases makes the sensitivity/specificity of microfluidic technology questionable compared to the laboratory-based methods. In lieu of developing a competing technology with the current state-of-the-art, it is recommended to develop more innovative platforms which can empower the existing technologies and provide court-proof results. This can further help overburdened forensic laboratories to accelerate analysis and testing.
3. Miniaturization of bulky peripherals: one of the other challenges which restricts the commercialization and final use of the microfluidic platforms for crime scene investigation is the need for bulky peripherals, e.g., pumps, optical detectors, power sources, etc. All the components must be miniaturized to achieve a fully portable platform. Research in this field has already been initiated to miniaturize peripheral set-ups and develop portable point-of-care (POC) devices [222]. It is suggested to consider a similar research direction to develop portable platforms for forensic applications.

## Figures and Tables

**Table 1:** Most commonly used presumptive blood tests based on chemical reaction (catalytic color change) along with corresponding pros and cons (inputs from references [20,21,22]).

Test Name	Reagent	Color Change	Pros	Cons
Luminol	5-Amino-2,3-dihydro-1,4-phthalazinedione	Colorless -> chemiluminescent blue light emission	Great sensitivity^1^ Great specificity^2^ Do not destroy DNA Can be reapplied Not carcinogenic	Must be used in near/complete darkness
Leuchomalachite green (LMG)	Reduced LMG	Colorless -> blue/green	As specific as Luminol	10-times less sensitivity than Luminol Can destroy DNA Carcinogenic
Kestle–Meyer (KL)	Reduced phenolphthalein	Colorless -> bright pink	Equal sensitivity to most of other tests	Extremely unspecific Can reduce amount of recoverable DNA Possible carcinogen
Hemastix^®^	3,3${}^{{}^{\prime }}$, 5,5${}^{{}^{\prime }}$-Tetramethylbenzidine (TMB)	Orange -> dark blue/green	Easy to transport/use Good sensitivity DNA can be recovered Not carcinogenic	Not very unspecific
Hemident^TM^	MacPhail’s reagent (leuchomalachite green)	Colorless -> blue/green	Specific Sensitive Self-contained chemical reaction	Can destroy DNA
Bluestar^©^	Similar as luminol	Colorless -> chemiluminescent blue	Good sensitivity Ease of preparation Long-lasting solution	Poor specificity Possible false positives Need for complete darkness

^1^ Sensitivity refers to the lowest detectable dilution of blood with distilled water [19]. Luminol is the most sensitive test with sensitivity as high as 1:1,000,000, followed by Hemastix^®^, KM, and LMG with the least sensitivity of 1:10,000 [22]. Sensitivity of Polilight^®^ is, respectively, 50,000 and 10 times less sensitive than luminol and LMG [21]. ^2^ Specificity refers to the chemical’s ability to accurately detect blood. Substance with similar appearance to blood or those containing oxidizing agent (peroxidase) are potential sources of false positive results [20].

**Table 2:** Commonly used drugs and the corresponding effect on the user.

Class of Drugs	Example	Effect
Central nervous system (CNS) depressants	Alcohol, barbiturates, gamma hydroxybutyrate (GHB), benzodiazepines	Slowing down the operations of brain and body
CNS stimulants	Amphetamines, cocaine, “crack” cocaine, methamphetamines (“crack”)	Over-stimulating the body by accelerating the heart rate and increasing blood pressure
Narcotic analgesics	Opium, heroin, morphine, methadone, oxycontin, codeine	Relieving pain by disabling brain’s perception of the pain, creating mood change and inducing euphoria
Psychotomimetics or hallucinogens	Lysergic acid diethylamide (LSD), methylenedioxymethamphetamine (MDMA) or ecstasy, psilocybin, mescaline	Mimicking the symptoms of psychosis, inducing delusions
Cannabis	Marijuana, synthetic cannabinoids	Causing psychological and physiological effects

**Table 3:** Three classes of commonly used explosives, the corresponding requirements, and the subsequent effects.

Type of Explosives	Example	Requirements and/or Effects
Low explosives: Combustible materials with reaction rates < speed of sound (3000 m/s) (subsonic)	Black powder (consisting of potassium nitrate, charcoal, and sulfur), smokeless powder	Upon reaction hot gases and inorganic residues are formed. Commonly contained in sealed casings to cause pressure build up.
High explosives: Reaction rates > speed of sound (detonation) without dependency on confinement	Primary explosives, e.g., mercury fulminate, lead azide, and triacetone triperoxide (TATP)	Sensitive to friction, shock, and heat
	Secondary explosives, e.g., TNT, nitroglycerin (NG), and cyclotrimethylenetrinitramene (RDX)	Increased stability with less sensitivity to heat or shock. Primary explosives are needed to provide large energy input for detonation
Blasting agents: Mixture of fuel and oxidizers prepared from fertilizers	Ammonium nitrate (AN) and urea nitrate (UN)	Less sensitive and require a booster to detonate

**Table 4:** A brief overview on the applications of microfluidic devices (inputs from reference [102]).

General Field	Specific Application	Opportunities and/or Advantages
Analytical platforms	Miniaturized counterpart of bulky columns for chromatography and mass spectrometry	Small concentration and volume of sample Fast results
Reaction and flow chemistry	Synthesis of materials through reactions occurring in microchannels	Industrial-scale material production
Point-of-care diagnostics	Diagnostics at place (home or remote areas)	No need for laboratory and trained personnel
Drug delivery	Invasive drug delivery using micro needles, inhalers or micropumps	Precise delivery of small amount of drug at targeted areas
Environmental testing	Inspection of water, air, or food quality to identify contaminants	Real-time monitoring Protecting health and safety of society
Biomedical research	Discovery and screening of new drugs Cell analysis Single cell sequencing	In situ synthesis and investigation of genes and proteins Biological mechanisms, metabolism, RNA, DNA

## Data Availability

Not applicable.

## Abbreviations

The following abbreviations are used in this manuscript.

ALS	Alternate light source
AN	Ammonium nitrate
BF	Body fluid
BFID	Body fluid identifictation
BFS	Body fluid screening
CBA	Cocaine-binding aptamer
CE	Capillary electrophoresis
CL	Chemiluminescence
CLSM	Confocal laser scanning microscope
CNC	Computer numerical control
CNS	Central nervous system
COC	Cyclic olefin copolymer
DE	Differential extraction
DNA	Deoxyribonucleic acid
DNB	1,3-dinitrobenzene
DNT	Dinitrotoluene
ECD	Electrochemical detection
ECL	Electrochemiluminescence
EDS	Energy dispersive spectroscopy
EDX	Energy dispersive X-ray analyzer
ELISA	Enzyme-linked immunosorbent assay
FBI	Federal Bureau of Investigation
FT-IR	Fourier transform infrared
GC–MS	Gas chromatography–mass spectrometry
GHB	Gamma hydroxybutyrate
Hb	Hemoglobin
HID	Human identification
HPLC	High-performance liquid chromatography
HSA	Heat-sensitive adhesive
HTN3	Histatin 3
IC	Ion chromatography
IMS	Ion mobility spectrometry
IR	Infrared
KL	Kestle–Meyer
LC–MS	Liquid chromatography–mass spectrometry
LLE	Liquid–liquid extraction
LMG	Leuchomalachite green
LOC	Lab-on-chip
LOD	Limit of detection
LSD	Lysergic acid diethylamide
MDMA	Methylenedioxymethamphetamine
ME	Microchip electrophoresis
MEKC	Micellar electrokinetic chromatography
MS	Mass spectrometry
NG	Nitroglycerin
PC	Polycarbonate
PCR	Polymerase chain reaction
PDMS	Polydimethylsiloxane
PeT	Polyethylene terephthalate
PETN	Pentaerythritol tetranitrate
PHP	Phenolphthalein
PMD	Portable microfluidic-based device
PMMA	Polymethylmethacrylate
POC	Point-of-care
PON	Point-of-need
PRM1	Protamine 1
PS	Polystyrene
PSA	Prostate-specific antigen
PU	Polyurethane
RDX	Cyclotrimethylenetrinitramene
RNA	Ribonucleic acid
RSID	Rapid stain identification
RT-PCR	Reverse transcription polymerase chain reaction
SAP	Seminal acid phosphatase
SDS	Sodium dodecyl sulfate
SEM	Scanning electron microscopy
SPE	Solid phase extraction
STATH	Statherin
STR	Short tandem repeat
TAK	Takayama
TATP	Triacetone triperoxide
TEI	Teichman’s
TLC	Thin layer chromatography
TMB	Tetramethylbenzidine
TNB	Trinitrobenzene
TNT	Trinitrotoluene
UN	Urea nitrate
UV	Ultraviolet
UV–Vis	Ultraviolet-visible
XRD	X-ray diffractometry
μPAD	Microfluidic paper-based analytical device
μPON	Microfluidic-based point-of-need
